# Supplementary material for: Higher Concentration of Dietary Selenium, Zinc, and Copper Complex Reduces Heat Stress-Associated Oxidative Stress and Metabolic Alteration in the Blood of Holstein and Jersey Steers
Source: Animals (Basel). 2022 Nov 10;12(22):3104. doi: 10.3390/ani12223104 (PMC9686896; doi:10.3390/ani12223104)
Supplement: Supplementary file 1 [file animals-12-03104-s001.zip › animals-1952937-supplementary.pdf]

animals

MDPI

Article

Higher concentration of dietary selenium, zinc, and copper complex reduces heat stress-associated oxidative stress and metabolic alteration in the blood of Holstein and Jersey steers

Supplementary Tables

Supplementary Table S1. Levels of 12 OAs, 16 FAs, 26 AAs, Kruskal–Wallis test, and VIP score of PLS–DA in Holstein serum.

Class	No.	Metabolites	Concentration (ng/μL, Mean ± SD)					Composition (%, Mean ± SD)					Kruskal Wallis test (P-value)	FDR[b]	VIP score
			Holstein Con	Holstein NM	Holstein HM	Normalized value[a]		Holstein Con	Holstein NM	Holstein HM	Normalized value[a]				
						NM	HM				NM	HM			
OA	1	Pyruvic acid	4.7 ± 1.3	5.3 ± 1.0	4.8 ± 0.24	1.13	1.01	1.9 ± 0.43	1.9 ± 0.13	1.8 ± 0.14	1.02	0.93	0.587	0.956	0.51
	2	Lactic acid	99.0 ± 25.6	95.9 ± 16.9	117.7 ± 6.4	0.97	1.19	39.8 ± 8.7	35.1 ± 2.4	43.2 ± 2.2	0.88	1.09	0.288	0.956	0.92
	3	Glycolic acid	83.6 ± 6.9	83.1 ± 12.6	82.0 ± 8.6	0.99	0.98	34.0 ± 3.4	30.7 ± 3.3	30.1 ± 2.8	0.90	0.88	0.393	0.956	1.81
	4	2-Hydroxybutyric acid	0.85 ± 0.14	0.78 ± 0.22	0.91 ± 0.073	0.92	1.07	0.35 ± 0.059	0.28 ± 0.054	0.33 ± < 0.01	0.82	0.97	0.561	0.956	0.29
	5	3-Hydroxypropionic acid	1.7 ± 0.50	2.4 ± 0.51	1.7 ± 0.71	1.39	0.97	0.70 ± 0.24	0.86 ± 0.077	0.59 ± 0.23	1.22	0.84	0.252	0.956	0.83
	6	3-Hydroxybutyric acid	24.7 ± 18.8	46.6 ± 20.9	30.1 ± 13.2	1.89	1.22	10.3 ± 8.2	16.4 ± 6.0	10.7 ± 4.0	1.60	1.04	0.561	0.956	0.68
	7	Succinic acid	1.5 ± 0.077	1.6 ± 0.33	1.5 ± 0.36	1.08	1.00	0.62 ± 0.019	0.60 ± 0.10	0.55 ± 0.10	0.98	0.89	0.670	0.956	1.19
	8	α-Ketoglutaric acid	1.0 ± 0.44	1.2 ± 0.28	1.3 ± 0.42	1.23	1.29	0.39 ± 0.15	0.47 ± 0.17	0.49 ± 0.19	1.19	1.23	0.875	0.956	0.65
	9	Malic acid	2.2 ± 1.1	1.5 ± 0.52	1.2 ± 0.16	0.67	0.54	0.9 ± 0.46	0.59 ± 0.31	0.44 ± 0.055	0.65	0.48	0.177	0.956	1.90
	10	2-Hydroxyglutaric acid	6.1 ± 1.3	6.5 ± 0.90	8.4 ± 1.1	1.06	1.37	2.5 ± 0.59	2.5 ± 0.79	3.1 ± 0.59	1.00	1.24	0.430	0.956	1.18
	11	Citric acid	3.1 ± 1.8	7.7 ± 1.3	5.9 ± 0.85	2.49	1.92	1.3 ± 0.76	2.9 ± 0.59	2.2 ± 0.18	2.22	1.67	0.079	0.956	1.68
	12	Hippuric acid	18.0 ± 5.6	20.0 ± 5.3	17.6 ± 3.4	1.11	0.98	7.3 ± 2.2	7.6 ± 2.3	6.5 ± 1.6	1.04	0.89	0.957	0.956	0.01
FA	13	Myristic acid ($C_{14:0}$)	2.4 ± 0.68	2.4 ± 0.66	2.8 ± 1.4	0.97	1.15	0.22 ± 0.039	0.22 ± 0.045	0.23 ± 0.090	1.03	1.08	0.875	0.956	0.89
	14	Palmitoleic acid ($C_{16:1}$)	4.0 ± 0.12	4.0 ± 0.91	5.2 ± 2.0	1.00	1.28	0.37 ± 0.081	0.38 ± 0.048	0.44 ± 0.12	1.02	1.17	0.957	0.956	0.31
	15	Palmitic acid ($C_{16:0}$)	140.5 ± 36.8	129.8 ± 21.0	138.0 ± 27.1	0.92	0.98	12.2 ± 1.1	12.3 ± 1.06	12.0 ± 0.46	1.00	0.98	0.670	0.956	1.68
	16	γ-Linolenic acid (γ-$C_{18:3}$)	4.7 ± 1.1	3.9 ± 0.19	4.0 ± 1.1	0.83	0.85	0.42 ± 0.012	0.38 ± 0.061	0.35 ± 0.066	0.92	0.85	0.587	0.956	1.51
	17	Linoleic acid ($C_{18:2}$)	155.7 ± 38.3	125.6 ± 4.5	138.5 ± 24.2	0.81	0.89	13.6 ± 0.49	12.1 ± 1.8	12.2 ± 1.0	0.89	0.89	0.733	0.956	0.79
	18	Oleic acid ($C_{18:1}$)	149.4 ± 18.0	146.1 ± 30.6	165.2 ± 38.7	0.98	1.11	13.5 ± 1.7	13.7 ± 1.5	14.3 ± 1.2	1.02	1.06	0.957	0.956	0.35
	19	α-Linolenic acid (α-$C_{18:3}$)	4.2 ± 0.64	3.6 ± 0.36	4.2 ± 0.92	0.86	0.98	0.38 ± 0.061	0.35 ± 0.080	0.36 ± 0.055	0.92	0.95	0.733	0.956	0.60
	20	Stearic acid ($C_{18:0}$)	215.8 ± 38.9	193.1 ± 27.3	213.4 ± 36.8	0.89	0.99	19.3 ± 1.7	18.3 ± 0.48	18.7 ± 1.2	0.95	0.97	0.875	0.956	0.40

	21	Arachidonic acid ($C_{20:4}$)	138.3 ± 21.4	135.1 ± 18.3	143.0 ± 13.8	0.98	1.03	12.4 ± 1.3	12.8 ± 0.93	12.7 ± 0.99	1.03	1.02	0.733	0.956	0.34
	22	Eicosadienoic acid ($C_{20:2}$)	1.4 ± 0.27	1.3 ± 0.12	1.4 ± 0.12	1.00	1.02	0.12 ± < 0.01	0.13 ± 0.014	0.12 ± 0.018	1.08	1.04	0.875	0.956	0.40
	23	Arachidic acid ($C_{20:0}$)	0.46 ± 0.095	0.41 ± 0.057	0.45 ± 0.10	0.90	0.99	0.040 ± < 0.01	0.039 ± < 0.01	0.039 ± < 0.01	0.97	0.97	0.837	0.956	0.84
	24	Docosatetraenoic acid ($C_{22:4}$)	306.1 ± 97.7	294.2 ± 42.5	312.9 ± 48.1	0.96	1.02	26.3 ± 3.1	28.0 ± 2.19	27.5 ± 0.38	1.06	1.04	0.430	0.956	1.04
	25	Docosapentaenoic acid ($C_{22:5}$)	7.6 ± 2.5	8.2 ± 0.88	8.2 ± 1.8	1.08	1.08	0.64 ± 0.10	0.78 ± 0.070	0.71 ± 0.086	1.21	1.11	0.875	0.956	0.70
	26	13-Methyltetradecanoic acid ($C_{14:0}$)	1.2 ± 0.13	1.1 ± 0.084	1.1 ± 0.27	0.91	0.99	0.11 ± 0.016	0.10 ± 0.010	0.10 ± < 0.01	0.96	0.94	0.733	0.956	0.07
	27	14-Methylpentadecanoic acid ($C_{15:0}$)	3.0 ± 0.60	2.8 ± 0.50	2.9 ± 0.36	0.95	1.00	0.26 ± 0.030	0.27 ± 0.055	0.26 ± 0.013	1.03	0.99	0.837	0.956	0.07
	28	16-Methylheptadecanoic acid ($C_{17:0}$)	0.64 ± 0.051	0.61 ± 0.063	0.66 ± 0.077	0.94	1.03	0.059 ± < 0.01	0.058 ± < 0.01	0.058 ± < 0.01	0.99	0.99	0.733	0.956	0.02
AA	29	Tryptophan	12.4 ± 2.7	12.2 ± 0.62	11.9 ± 1.7	0.99	0.96	4.3 ± 0.62	4.0 ± 0.071	4.1 ± 0.61	0.94	0.96	0.837	0.956	0.36
	30	Phenylalanine	8.2 ± 0.75	8.4 ± 0.084	8.6 ± 0.79	1.03	1.05	2.9 ± 0.023	2.8 ± 0.07	2.9 ± 0.21	0.98	1.03	0.430	0.956	0.84
	31	Tyrosine	4.0 ± 0.71	4.3 ± 0.14	3.7 ± 0.32	1.06	0.91	1.4 ± 0.16	1.4 ± 0.071	1.3 ± 0.15	1.01	0.91	0.561	0.956	1.15
	32	Leucine	16.5 ± 2.1	17.2 ± 0.77	18.3 ± 1.0	1.04	1.11	5.7 ± 0.26	5.7 ± 0.38	6.3 ± 0.56	0.99	1.11	0.430	0.956	1.58
	33	Methionine	2.9 ± 0.54	3.3 ± 0.29	2.9 ± 0.24	1.13	0.97	1.0 ± 0.11	1.1 ± 0.12	0.99 ± 0.10	1.08	0.97	0.561	0.956	0.30
	34	Isoleucine	9.4 ± 1.2	10.2 ± 0.26	10.3 ± 0.83	1.09	1.10	3.2 ± 0.16	3.4 ± 0.14	3.6 ± 0.34	1.04	1.10	0.491	0.956	1.57
	35	Valine	20.5 ± 3.4	20.6 ± 1.7	21.1 ± 2.6	1.00	1.03	7.1 ± 0.63	6.8 ± 0.58	7.3 ± 1.0	0.96	1.03	0.837	0.956	0.29
	36	Pipecolic acid	0.055 ± < 0.01	0.055 ± < 0.01	0.061 ± < 0.01	1.00	1.11	0.019 ± < 0.01	0.018 ± < 0.01	0.021 ± < 0.01	0.94	1.09	0.252	0.956	1.39
	37	Glutamic acid	3.9 ± 0.37	4.2 ± 0.29	4.2 ± 0.65	1.09	1.09	1.3 ± 0.022	1.4 ± 0.14	1.4 ± 0.17	1.04	1.07	0.957	0.956	1.02
	38	α-Aminobutyric acid	1.3 ± 0.22	1.4 ± 0.090	1.3 ± 0.11	1.09	1.04	0.44 ± 0.041	0.46 ± 0.041	0.46 ± 0.058	1.04	1.04	0.430	0.956	0.53
	39	Proline	5.7 ± 0.39	6.7 ± 0.83	6.2 ± 0.52	1.18	1.10	2.0 ± 0.088	2.2 ± 0.20	2.1 ± 0.011	1.11	1.08	0.113	0.956	1.87
	40	Hydroxyproline	1.4 ± 0.23	1.5 ± 0.12	1.4 ± 0.16	1.02	0.98	0.51 ± 0.12	0.48 ± 0.049	0.49 ± 0.073	0.95	0.96	0.957	0.956	0.19
	41	Threonine	1.6 ± 0.57	1.7 ± 0.34	1.5 ± 0.33	1.12	0.96	0.53 ± 0.16	0.57 ± 0.11	0.51 ± 0.080	1.08	0.96	0.837	0.956	0.05
	42	Alanine	17.2 ± 1.6	20.3 ± 1.2	22.9 ± 1.8	1.18	1.33	6.0 ± 0.45	6.7 ± 0.45	7.9 ± 0.32	1.11	1.31	0.051	0.956	3.09
	43	Serine	5.2 ± 0.38	6.4 ± 0.86	5.3 ± 0.65	1.24	1.02	1.8 ± 0.036	2.1 ± 0.22	1.8 ± 0.10	1.17	1.00	0.061	0.956	0.02
	44	Creatine	34.3 ± 1.8	33.5 ± 5.1	34.0 ± 0.48	0.98	0.99	12.0 ± 0.52	11.1 ± 1.8	11.7 ± 0.79	0.92	0.98	0.837	0.956	0.23
	45	Glutamine	91.9 ± 4.1	97.3 ± 12.2	88.3 ± 17.8	1.06	0.96	32.2 ± 1.54	32.0 ± 3.1	30.1 ± 3.91	0.99	0.94	0.837	0.956	1.10
	46	Creatinine	7.7 ± 0.88	8.5 ± 0.68	7.8 ± 0.30	1.10	1.01	2.7 ± 0.55	2.8 ± 0.13	2.7 ± 0.27	1.03	0.99	0.733	0.956	0.10
	47	Asparagine	2.8 ± 0.36	3.4 ± 0.16	2.9 ± 0.60	1.21	1.01	0.98 ± 0.050	1.1 ± 0.016	0.97 ± 0.13	1.15	0.99	0.288	0.956	0.16
	48	Citrulline	9.7 ± 2.1	9.8 ± 2.0	8.9 ± 2.0	1.01	0.92	3.3 ± 0.54	3.2 ± 0.77	3.1 ± 0.80	0.97	0.92	0.957	0.956	0.62
	49	1-Methylhistidine	0.64 ± 0.12	0.80 ± 0.041	0.60 ± 0.051	1.26	0.94	0.23 ± 0.064	0.3 ± 0.022	0.21 ± 0.016	1.17	0.90	0.288	0.956	0.48
	50	Histidine	9.7 ± 0.93	9.3 ± 1.0	8.9 ± 0.95	0.96	0.92	3.4 ± 0.13	3.1 ± 0.24	3.1 ± 0.18	0.91	0.91	0.288	0.956	1.80
	51	3-Methylhistidine	1.1 ± 0.17	1.2 ± 0.19	1.0 ± 0.093	1.11	0.99	0.38 ± 0.097	0.39 ± 0.060	0.36 ± 0.039	1.03	0.96	0.875	0.956	0.14
	52	Lysine	4.7 ± 1.5	4.7 ± 0.47	4.4 ± 0.65	0.99	0.93	1.6 ± 0.41	1.5 ± 0.21	1.5 ± 0.19	0.96	0.94	0.957	0.956	0.29
	53	Ornithine	4.9 ± 0.89	5.3 ± 0.48	4.9 ± 0.49	1.10	1.00	1.7 ± 0.19	1.8 ± 0.17	1.7 ± 0.10	1.05	0.99	0.875	0.956	0.01
	54	Arginine	9.5 ± 1.3	11.0 ± 1.1	9.7 ± 1.0	1.16	1.02	3.3 ± 0.20	3.6 ± 0.49	3.3 ± 0.087	1.10	1.01	0.875	0.956	0.20

[a] Values normalized to the corresponding control composition mean values
[b] False discovery rate by Fisher least significant difference method
Con: only TMR (without mineral supplementation), NM: TMR + NRC recommended concentration of mineral supplementation (Se 0.1 ppm + Zn 30 ppm + Cu 10 ppm)/kg DM and HM: TMR + higher than recommended concentration of mineral supplementation (Se 3.5 ppm + Zn 350 ppm + Cu 28 ppm)/kg DM.

Supplementary Table S2. Levels of 12 OAs, 16 FAs, 26 AAs, Kruskal–Wallis test, and VIP score of PLS–DA in Jersey serum.

Class	No.	Metabolites	Concentration (ng/μL, Mean ± SD)					Composition (%, Mean ± SD)					Kruskal Wallis test (P-value)	FDR[b]	VIP score
			Jersey Con	Jersey NM	Jersey HM	Normalized value[a]		Jersey Con	Jersey NM	Jersey HM	Normalized value[a]				
						NM	HM				NM	HM			
	1	Pyruvic acid	4.0 ± 0.15	4.7 ± 0.16	6.0 ± 1.4	1.19	1.51	1.7 ± 0.11	1.9 ± 0.037	2.0 ± 0.25	1.16	1.24	0.193	0.956	2.10
	2	Lactic acid	96.2 ± 9.7	90.5 ± 6.5	140.9 ± 50.9	0.94	1.46	39.5 ± 1.2	36.4 ± 2.4	45.6 ± 4.0	0.92	1.15	0.027	0.956	1.81
	3	Glycolic acid	87.9 ± 8.8	88.8 ± 5.4	111.6 ± 49.2	1.01	1.27	36.4 ± 4.4	35.7 ± 1.46	35.2 ± 2.6	0.98	0.97	0.670	0.956	0.72
	4	2-Hydroxybutyric acid	0.79 ± 0.070	0.79 ± 0.17	1.3 ± 0.57	1.01	1.62	0.32 ± 0.022	0.32 ± 0.066	0.43 ± 0.21	0.98	1.33	0.957	0.956	0.77
	5	3-Hydroxypropionic acid	1.3 ± 0.39	2.4 ± 0.39	1.6 ± 0.79	1.82	1.23	0.53 ± 0.13	0.9 ± 0.13	0.51 ± 0.13	1.79	0.96	0.066	0.956	0.22
OA	6	3-Hydroxybutyric acid	22.7 ± 18.6	29.4 ± 2.5	19.3 ± 13.2	1.29	0.85	8.8 ± 6.7	11.8 ± 0.89	5.9 ± 2.7	1.34	0.67	0.252	0.956	0.65
	7	Succinic acid	1.4 ± 0.21	1.3 ± 0.16	1.6 ± 0.69	0.87	1.07	0.59 ± 0.061	0.51 ± 0.066	0.49 ± 0.077	0.85	0.82	0.252	0.956	2.09
	8	α-Ketoglutaric acid	1.2 ± 0.091	1.3 ± 0.27	1.5 ± 0.50	1.12	1.26	0.48 ± 0.087	0.52 ± 0.12	0.54 ± 0.25	1.08	1.12	0.957	0.956	0.05
	9	Malic acid	1.5 ± 0.51	1.5 ± 0.21	1.5 ± 0.94	0.99	1.01	0.63 ± 0.24	0.59 ± 0.073	0.44 ± 0.11	0.95	0.71	0.430	0.956	1.21
	10	2-Hydroxyglutaric acid	8.9 ± 1.0	7.7 ± 1.0	9.8 ± 3.2	0.87	1.10	3.7 ± 0.77	3.1 ± 0.46	3.3 ± 0.88	0.84	0.88	0.587	0.956	1.02
	11	Citric acid	2.9 ± 1.7	5.1 ± 1.3	2.1 ± 1.8	1.77	0.73	1.3 ± 0.81	2.1 ± 0.57	1.0 ± 0.93	1.66	0.77	0.393	0.956	0.91
	12	Hippuric acid	14.7 ± 1.0	15.1 ± 6.1	11.8 ± 5.1	1.02	0.80	6.2 ± 1.0	6.1 ± 2.5	4.7 ± 3.3	0.99	0.76	0.670	0.956	0.81
	13	Myristic acid ($C_{14:0}$)	1.5 ± 0.19	1.6 ± 0.33	1.7 ± 0.51	1.05	1.16	0.19 ± < 0.01	0.17 ± 0.019	0.23 ± 0.076	0.92	1.22	0.733	0.956	1.61
	14	Palmitoleic acid ($C_{16:1}$)	2.9 ± 0.57	3.2 ± 0.30	3.8 ± 1.2	1.12	1.32	0.36 ± 0.010	0.36 ± 0.029	0.49 ± 0.15	1.00	1.38	0.561	0.956	1.69
	15	Palmitic acid ($C_{16:0}$)	92.3 ± 16.0	104.9 ± 12.3	96.0 ± 6.7	1.14	1.04	11.4 ± 0.14	11.5 ± 0.63	12.4 ± 1.0	1.01	1.09	0.561	0.956	1.34
	16	γ-Linolenic acid (γ-$C_{18:3}$)	3.2 ± 1.1	3.5 ± 2.1	1.9 ± 0.92	1.08	0.57	0.39 ± 0.068	0.36 ± 0.19	0.25 ± 0.14	0.91	0.64	0.288	0.956	1.79
	17	Linoleic acid ($C_{18:2}$)	126.3 ± 22.9	134.9 ± 29.8	98.9 ± 16.6	1.07	0.78	15.6 ± 1.1	14.5 ± 1.2	12.9 ± 2.7	0.93	0.83	0.733	0.956	0.48
	18	Oleic acid ($C_{18:1}$)	92.9 ± 21.0	105.1 ± 13.5	95.2 ± 19.9	1.13	1.03	11.6 ± 2.6	11.5 ± 1.0	12.3 ± 2.4	0.99	1.06	0.837	0.956	1.20
	19	α-Linolenic acid (α-$C_{18:3}$)	3.4 ± 0.71	3.7 ± 1.0	2.8 ± 0.57	1.08	0.83	0.42 ± 0.031	0.39 ± 0.053	0.37 ± 0.10	0.94	0.88	0.288	0.956	1.41
	20	Stearic acid ($C_{18:0}$)	152.2 ± 22.2	161.2 ± 21.7	137.8 ± 2.5	1.06	0.91	18.9 ± 0.9	17.6 ± 0.61	17.9 ± 1.2	0.93	0.94	0.957	0.956	0.46
FA	21	Arachidonic acid ($C_{20:4}$)	103.1 ± 24.4	113.6 ± 16.1	102.5 ± 13.6	1.10	0.99	12.7 ± 2.5	12.4 ± 0.13	13.3 ± 2.2	0.97	1.05	0.875	0.956	1.09
	22	Eicosadienoic acid ($C_{20:2}$)	1.9 ± 0.87	1.6 ± 0.24	1.3 ± 0.24	0.81	0.67	0.26 ± 0.15	0.18 ± 0.048	0.17 ± 0.026	0.68	0.65	0.079	0.956	1.08
	23	Arachidic acid ($C_{20:0}$)	0.30 ± 0.028	0.45 ± 0.12	0.26 ± 0.013	1.47	0.84	0.038 ± < 0.01	0.048 ± < 0.01	0.033 ± < 0.01	1.25	0.87	0.561	0.956	0.32
	24	Docosatetraenoic acid ($C_{22:4}$)	218.2 ± 47.6	272.6 ± 42.5	222.1 ± 57.8	1.25	1.02	26.8 ± 2.27	29.7 ± 1.31	28.3 ± 6.0	1.11	1.06	0.837	0.956	0.11
	25	Docosapentaenoic acid ($C_{22:5}$)	6.5 ± 1.6	7.5 ± 1.1	6.9 ± 2.8	1.15	1.07	0.80 ± 0.17	0.81 ± 0.023	0.88 ± 0.32	1.01	1.09	0.837	0.956	0.98
	26	13-Methyltetradecanoic acid ($C_{14:0}$)	0.84 ± 0.17	0.91 ± 0.33	0.69 ± 0.35	1.09	0.83	0.10 ± < 0.01	0.10 ± 0.025	0.092 ± 0.051	0.93	0.90	0.670	0.956	1.14
	27	14-Methylpentadecanoic acid ($C_{15:0}$)	2.2 ± 0.40	2.6 ± 0.79	1.7 ± 0.61	1.21	0.80	0.27 ± < 0.01	0.28 ± 0.055	0.23 ± 0.089	1.05	0.85	0.875	0.956	0.53
	28	16-Methylheptadecanoic acid ($C_{17:0}$)	0.53 ± 0.088	0.58 ± 0.14	0.49 ± 0.017	1.08	0.92	0.066 ± < 0.01	0.062 ± < 0.01	0.064 ± < 0.01	0.94	0.97	0.491	0.956	1.12
	29	Tryptophan	12.0 ± 3.2	10.3 ± 2.4	10.0 ± 1.8	0.85	0.83	4.0 ± 0.71	4.0 ± 0.33	3.7 ± 0.10	0.99	0.93	0.875	0.956	0.61
	30	Phenylalanine	9.3 ± 0.65	8.4 ± 0.44	9.5 ± 0.66	0.91	1.03	3.2 ± 0.26	3.4 ± 0.82	3.7 ± 0.91	1.09	1.17	0.957	0.956	0.95
	31	Tyrosine	4.7 ± 0.29	3.8 ± 0.82	3.8 ± 0.80	0.82	0.82	1.6 ± 0.22	1.5 ± 0.15	1.4 ± 0.13	0.93	0.89	0.733	0.956	1.23
AA	32	Leucine	20.0 ± 2.1	18.7 ± 4.3	20.4 ± 3.0	0.93	1.02	6.8 ± 0.63	7.3 ± 1.1	7.7 ± 0.57	1.06	1.13	0.561	0.956	1.43
	33	Methionine	3.8 ± 0.39	3.1 ± 0.65	3.5 ± 0.35	0.82	0.92	1.3 ± 0.17	1.2 ± 0.056	1.3 ± 0.086	0.93	1.02	0.393	0.956	0.51
	34	Isoleucine	12.9 ± 1.5	10.5 ± 2.1	11.7 ± 1.7	0.81	0.90	4.4 ± 0.58	4.1 ± 0.55	4.4 ± 0.091	0.93	0.99	0.837	0.956	0.12

35	Valine	24.5 ± 3.4	21.1 ± 4.9	22.8 ± 4.5	0.86	0.93	8.3 ± 0.58	8.2 ± 1.1	8.5 ± 0.95	0.99	1.03	0.561	0.956	0.41
36	Pipecolic acid	0.067 ± 0.010	0.062 ± < 0.01	0.062 ± < 0.01	0.93	0.94	0.023 ± < 0.01	0.025 ± < 0.01	0.024 ± < 0.01	1.10	1.05	0.957	0.956	0.39
37	Glutamic acid	3.9 ± 0.17	3.7 ± 0.63	3.7 ± 0.42	0.93	0.93	1.3 ± 0.11	1.4 ± 0.068	1.4 ± 0.10	1.06	1.03	0.670	0.956	0.74
38	α-Aminobutyric acid	1.2 ± 0.11	1.2 ± 0.46	0.90 ± 0.53	1.01	0.77	0.40 ± 0.016	0.44 ± 0.12	0.35 ± 0.19	1.11	0.87	0.837	0.956	1.01
39	Proline	7.1 ± 0.45	5.5 ± 1.0	6.2 ± 1.4	0.78	0.88	2.4 ± 0.18	2.2 ± 0.19	2.3 ± 0.23	0.89	0.96	0.252	0.956	0.52
40	Hydroxyproline	1.5 ± 0.37	1.3 ± 0.26	1.2 ± 0.29	0.88	0.81	0.50 ± 0.074	0.5 ± 0.071	0.45 ± 0.044	1.04	0.90	0.393	0.956	1.00
41	Threonine	2.7 ± 0.73	2.2 ± 1.0	2.5 ± 0.92	0.82	0.93	0.91 ± 0.16	0.81 ± 0.26	0.92 ± 0.21	0.90	1.01	0.957	0.956	0.03
42	Alanine	19.4 ± 1.1	15.8 ± 2.2	17.7 ± 1.9	0.82	0.91	6.6 ± 0.60	6.2 ± 0.49	6.7 ± 0.66	0.94	1.02	0.670	0.956	0.31
43	Serine	5.5 ± 0.77	4.5 ± 1.2	4.5 ± 1.2	0.81	0.82	1.9 ± 0.089	1.7 ± 0.19	1.7 ± 0.21	0.93	0.89	0.393	0.956	1.61
44	Creatine	25.8 ± 3.0	20.5 ± 3.2	23.6 ± 1.4	0.79	0.91	8.8 ± 0.68	8.0 ± 0.61	9.1 ± 1.5	0.92	1.03	0.430	0.956	0.30
45	Glutamine	90.8 ± 14.2	80.6 ± 26.3	81.1 ± 17.3	0.89	0.89	30.7 ± 2.4	30.6 ± 4.1	30.1 ± 1.9	1.00	0.98	0.733	0.956	0.16
46	Creatinine	9.7 ± 1.0	9.2 ± 1.4	8.8 ± 1.5	0.95	0.91	3.3 ± 0.22	3.8 ± 1.4	3.3 ± 0.44	1.16	1.01	0.957	0.956	0.07
47	Asparagine	3.3 ± 0.58	2.7 ± 0.86	2.8 ± 0.89	0.82	0.84	1.1 ± 0.12	1.0 ± 0.12	1.0 ± 0.18	0.92	0.91	0.670	0.956	1.02
48	Citrulline	7.9 ± 3.0	7.1 ± 2.4	5.9 ± 1.8	0.89	0.74	2.6 ± 0.80	2.7 ± 0.57	2.1 ± 0.38	1.03	0.83	0.587	0.956	0.73
49	1-Methylhistidine	0.85 ± 0.13	0.90 ± 0.35	0.87 ± 0.45	1.05	1.02	0.29 ± 0.011	0.38 ± 0.22	0.32 ± 0.14	1.34	1.12	0.957	0.956	0.03
50	Histidine	8.9 ± 1.5	7.7 ± 1.8	7.3 ± 1.9	0.86	0.82	3.0 ± 0.17	3.0 ± 0.16	2.7 ± 0.35	0.99	0.90	0.561	0.956	1.54
51	3-Methylhistidine	1.0 ± 0.061	1.1 ± 0.35	1.0 ± 0.18	1.10	0.99	0.34 ± 0.050	0.47 ± 0.25	0.38 ± 0.089	1.38	1.11	0.957	0.956	0.40
52	Lysine	4.6 ± 1.3	4.0 ± 1.2	3.9 ± 1.0	0.87	0.86	1.5 ± 0.29	1.5 ± 0.22	1.4 ± 0.15	1.00	0.95	0.875	0.956	0.22
53	Ornithine	4.7 ± 1.3	4.7 ± 1.6	4.2 ± 1.0	0.99	0.90	1.6 ± 0.34	1.7 ± 0.30	1.6 ± 0.15	1.11	0.99	0.875	0.956	0.11
54	Arginine	9.2 ± 1.0	9.4 ± 1.4	8.7 ± 1.2	1.02	0.95	3.1 ± 0.051	3.7 ± 0.53	3.3 ± 0.068	1.19	1.05	0.051	0.956	0.76

[a] Values normalized to the corresponding control composition mean values

[b] False discovery rate by Fisher least significant difference method

Con: only TMR (without mineral supplementation), NM: TMR + NRC recommended concentration of mineral supplementation (Se 0.1 ppm + Zn 30 ppm + Cu 10 ppm)/kg DM and HM: TMR + higher than recommended concentration of mineral supplementation (Se 3.5 ppm + Zn 350 ppm + Cu 28 ppm)/kg D
